# The Effects of BMP-2, miR-31, miR-106a, and miR-148a on Osteogenic Differentiation of MSCs Derived from Amnion in Comparison with MSCs Derived from the Bone Marrow

**DOI:** 10.1155/2017/7257628

**Published:** 2017-11-19

**Authors:** Sirikul Manochantr, Kulisara Marupanthorn, Chairat Tantrawatpan, Pakpoom Kheolamai, Duangrat Tantikanlayaporn, Prakasit Sanguanjit

**Affiliations:** ^1^Division of Cell Biology, Department of Preclinical Sciences, Faculty of Medicine, Thammasat University, Pathumthani 12120, Thailand; ^2^Center of Excellence in Stem Cell Research, Thammasat University, Pathumthani 12120, Thailand; ^3^Department of Orthopedics, Faculty of Medicine, Thammasat University, Pathumthani 12120, Thailand

## Abstract

Mesenchymal stromal cells (MSCs) offering valuable anticipations for the treatment of degenerative diseases. They can be found in many tissues including amnion. MSCs from amnion (AM-MSCs) can differentiate into osteoblast similar to that of bone marrow-derived MSCs (BM-MSCs). However, the ability is not much efficient compared to BM-MSCs. This study aimed to examine the effects of BMP-2 and miRNAs on osteogenic differentiation of AM-MSCs compared to those of BM-MSCs. The osteogenic differentiation capacity after miRNA treatment was assessed by ALP expression, ALP activity, and osteogenic marker gene expression. The results showed that the osteogenic differentiation capacity increased after BMP-2 treatment both in AM-MSCs and BM-MSCs. MiR-31, miR-106a, and miR-148a were downregulated during the osteogenic differentiation. After transfection with anti-miRNAs, ALP activity and osteogenic genes were increased over the time of differentiation. The data lead to the potential for using AM-MSCs as an alternative source for bone regeneration. Moreover, the information of miRNA expression and function during osteogenic differentiation may be useful for the development of new therapeutics or enhanced an *in vitro* culture technique required for stem cell-based therapies in the bone regeneration.

## 1. Introduction

Mesenchymal stromal cells (MSCs) have increased an important potential in regenerative medicine due to their multipotential differentiation [1]. Nowadays, MSCs can be isolated from many tissues including bone marrow, amnion, placenta, and umbilical cord [1–3]. A previous study reported the variation in differentiation potential, especially the osteogenic differentiation, of MSCs which were derived from different tissues [4]. A preliminary study showed that MSCs derived from amnion (AM-MSCs) could differentiate into osteoblast; nevertheless, the differentiation capacity is not steady.

Bone morphogenetic proteins (BMPs), a powerful morphogens, could determine a lineage differentiation by activating specific transcriptional pathway [5]. Specifically, BMP-2 has been described as a morphogen for bone regeneration [6, 7]. The benefit of BMP-2 for bone tissue regeneration has been extensively studied, mostly in bone marrow-derived MSCs (BM-MSCs) [8–11]. However, the effect of BMP-2 for enhancing osteogenic differentiation ability of AM-MSCs is not completely studied.

In addition, microRNAs (miRNAs) have been reported as key regulators in almost every cellular process including the differentiation of stem cells [12, 13]. These small noncoding RNAs regulate gene expression mainly by suppressing the expression of specific transcription factors through binding the 3′ untranslated region of their target mRNAs [14]. Over the past few years, there are an increasing number of researches addressing the involvement of miRNAs in osteogenic differentiation and bone development. Various miRNAs have been reported to affect the fate of bone differentiation including miR-31, miR-106a, and miR-148a [15]. These miRNAs regulated the expression of RUNX-2 which is considered as the first master transcription factor responsible for the acquisition of osteochondroblastic characteristics [16]. Nevertheless, the relation between miRNA expression and the osteogenic differentiation potential of AM-MSCs remains elusive. Therefore, this study aimed to examine the effects of BMP-2 and the influence of miRNAs on osteogenic differentiation of AM-MSCs compared to those of BM-MSCs. The data obtained provide new insights into the effects of BMP-2 and miRNAs on osteogenic differentiation of AM-MSCs and BM-MSCs which lead to the feasibility for using miRNA as a modulator for bone regeneration in the future.

## 2. Materials and Methods

### 2.1. Cell Isolation and Culture

This protocol was approved by the Human Ethics Committee of Thammasat University No. 1 (Faculty of Medicine). All volunteers (*n* = 4) were <60 years of age and had no past history of infectious diseases. A 5–10 ml of bone marrow was harvested, and mononuclear cells were isolated using Ficoll-Hypaque solution (Sigma-Aldrich, USA). The cells were then cultured in Dulbecco's Modified Eagle's Medium (DMEM; GibcoBRL, USA) supplemented with 10% fetal bovine serum (FBS; Invitrogen, USA), 2 mM L-glutamine (GibcoBRL, USA), 100 U/ml penicillin, and 100 *μ*g/ml streptomycin (GibcoBRL, USA). Nonadherent cells were removed, and fresh medium was added at day 4. Subsequently, the medium was changed every 3 days. The expanded fibroblast-like cells which reached 80% confluence were subcultured using 0.25% trypsin-EDTA (GibcoBRL, USA) and replated at a density of 1 × 10^4^ cells/cm^2^.

The amniotic tissues from 4 donors were chopped into small pieces and digested with 1.6 mg/ml collagenase XI (Sigma-Aldrich, USA) and 200 mg/ml deoxyribonuclease I (Sigma-Aldrich, USA) at 37°C for 4 h. After washing, the cells were cultured with completed medium similar to BM-MSCs. The cells were refed every 3 days until getting the colonies of fibroblast-like cells. The cells were expanded in monolayer and subcultured using 0.25% trypsin-EDTA.

### 2.2. Flow Cytometric Analysis of Cultured MSCs

Immunophenotype of cultured MSCs was examined at passage 3–5 using flow cytometry. Briefly, 5 × 10^5^ MSCs were harvested and resuspended with 50 *μ*l of phosphate buffer saline (PBS). The cells were subsequently incubated with 5 *μ*l of antibodies including PE-CD34 antibody (BioLegend, USA), FITC-CD45 antibody (BioLegend, USA), PE-CD73 antibody (BioLegend, USA), FITC-CD90 antibody (BioLegend, USA), and PE-CD105 antibody (BD Bioscience, USA), for 30 min at 4°C. After washing with PBS, the cells were fixed with 1% paraformaldehyde for 15 min. At least 10,000 labeled cells were acquired and analyzed using flow cytometry (FACScalibur™, Becton Dickinson, USA) and CellQuest® software (Becton Dickinson, USA).

### 2.3. The Differentiation Assay

MSCs from both bone marrow and amnion were subjected to adipogenic and osteogenic differentiation at passage 3–5. For adipogenic differentiation, 7.5 × 10^4^ MSCs were seeded in 35 mm^2^ dishes containing adipogenic differentiation medium (DMEM supplemented with 10% FBS, 100 U/ml penicillin, 100 *μ*g/ml streptomycin, 0.5 mM isobutyl-methylxanthine (Sigma-Aldrich, USA), 1 *μ*M dexamethasone, 10 *μ*M insulin (Sigma-Aldrich, USA), 25 mM glucose, and 100 *μ*M indomethacin (Sigma-Aldrich, USA)). The medium was changed every 4 days. After 21 days, the cells were fixed in 10% buffered formalin for 30 min at room temperature, washed with PBS, and incubated with 2% Oil Red O (Sigma-Aldrich, USA) for 1 h. The cells were washed with distilled H_2_O and observed under an inverted microscope (Nikon TS100, Japan).

For osteogenic differentiation, 4.5 × 10^4^ MSCs were seeded in 35 mm^2^ dishes containing osteogenic differentiation medium (DMEM supplemented with 10% FBS, 100 U/ml penicillin, 100 *μ*g/ml streptomycin, 100 nM dexamethasone, and 50 *μ*g/ml ascorbic acid (Sigma-Aldrich, USA)). On day 7 of induction, 10 mM *β*-glycerophosphate (Sigma-Aldrich, USA) was added. To detect mineralized matrix, the cells were subject to Alizarin red staining (Sigma-Aldrich, USA). After removing the medium, cells were rinsed with PBS and fixed with 4% paraformaldehyde for 10 min at 4°C. The cells were washed twice with distilled water and stained with 40 mM Alizarin red (pH 4.2) for 30 min at room temperature. Control cultures without the differentiation stimuli were carried out in parallel to the experiments and stained in the same manner.

### 2.4. The Effect of BMP-2 on Osteogenic Differentiation of MSCs

To examine the effect of BMP-2 on osteogenic differentiation of MSCs, 9.5 × 10^3^ MSCs at passage 4 were cultured in 24-well plate (Corning, USA) with osteogenic differentiation medium supplemented with 100 ng/ml of BMP-2 (R&D systems, USA) for 3, 7, 14, 21, and 28 days. The osteogenic differentiation was measured by alkaline phosphatase staining using 5-bromo-4-chloro-3-indolylphosphate/nitro blue tetrazolium (BCIP/NBT; Sigma-Aldrich, USA). Briefly, the cultured cells were washed with PBS and fixed with 4% paraformaldehyde for 5 min at 4°C. Then, BCIP®/NBT® liquid substrate (Sigma-Aldrich, USA) was added and incubated for 30 min at a room temperature. The cells were wash twice with distilled water and observed under inverted microscope (Nikon TS100, Japan). MSCs cultured with complete medium and osteogenic differentiation medium were used as controls.

### 2.5. The Measurement of Alkaline Phosphatase Activity

The ALP activity was determined using SensoLyte® pNPP Alkaline Phosphatase Assay Kit (Anaspec, Inc., USA) according to the manufacturer's instruction. Briefly, MSCs were cultured with osteogenic differentiation medium with or without BMP-2 for 3, 7, 14, 21, and 28 days. Afterward, the cells were washed twice and permeabilized using 0.2% Triton X-100. The cells were subsequently incubated with substrate solution, p-nitrophenylphosphate (pNPP), for 45 min at room temperature. After stop reaction, the absorbance was measured at 405 nm. The total protein concentration (mg/ml) was determined using bicinchoninic acid (BCA) assay kit (Sigma-Aldrich, USA). ALP levels were normalized against the total protein content, and the ALP activities were expressed as ng/mg protein. MSCs cultured with complete medium were used as a control.

### 2.6. Quantitative Real-Time PCR (qRT-PCR) for Osteogenic Gene Expression

To examine the osteogenic gene expression, MSCs cultured with osteogenic differentiation medium with or without BMP-2 for 3, 7, 14, 21, and 28 days were harvested using 0.25% trypsin-EDTA. Total RNA was extracted from each sample using TRIzol® solution (Invitrogen, USA). The first-strand complementary DNA was synthesized using Superscript® III Reverse Transcriptase (Invitrogen, USA). The qRT-PCR was performed using SYBR® Green PCR Master Mix (Invitrogen, USA) and the ABI 7500 Real-time PCR System (Applied Biosystems, USA). The relative mRNA level was expressed as fold changes relative to untreated controls after normalization to the expression of glyceraldehyde-3-phosphate dehydrogenase (*GAPDH*). Primers were specified in Table 1.

### 2.7. Determination of the Expression Levels of MicroRNAs Involved in Osteogenic Differentiation

The expression levels of microRNAs involved in osteogenic differentiation, miR-31, miR-106a, and miR-148a, were examined using qRT-PCR. Briefly, MSCs were cultured in osteogenic differentiation medium with or without 100 ng/ml BMP-2 for 3, 7, 14, 21, and 28 days. Then, total RNA was extracted from the cultured MSCs using TRIzol reagent (Invitrogen, USA). MicroRNAs were reverse transcribed into cDNA using TaqMan® microRNA Reverse Transcription Kit (Applied Biosystems, USA). The reverse transcription was carried in a MyCycler Thermal Cycler (Bio-Rad, USA). The reaction mixtures were incubated at 16°C for 30 min, then at 42°C for 30 min, and then inactivated at 85°C for 5 min. The qRT-PCR samples were then prepared using TaqMan Universal PCR Master Mix II (2X) (Applied Biosystems, USA). The miRNA-specific primers (Table 2) were included in TaqMan microRNA Inventoried Assays (Applied Biosystems, USA). The assays used in the study included miR-31-5p, miR-106a-5p, and miR-148a-5p. For quantitative calibration and normalization, the expression of U6 was used as an endogenous control.

### 2.8. Transient Transfections with miRNA Inhibitors

To explore the effect of miRNAs (miR-31, miR-106a, and miR-148a) on osteogenic differentiation potential of MSCs, miRNA inhibitors were transient transfected into MSCs using Lipofectamine 3000 transfection reagent (Invitrogen, USA). Briefly, 10 nM of each miRNA inhibitors diluted in osteogenic differentiation medium was mixed with Lipofectamine 3000 transfection reagent and incubated for 10 min at room temperature. Then, the transfection complexes were added into the cell cultures. MSCs transfected with 10 nM FAM-labeled miRNA negative control #1 were used as a negative control. After 3, 7, 14, and 21 days of incubation, total RNA was extracted and miRNAs expression levels were determined using qRT-PCR. The alkaline phosphatase activity assay was performed to determine the osteogenic differentiation potential. In addition, the expression of genes involved in osteogenic differentiation was also studied.

### 2.9. Statistical Analysis

The experimental statistics presented in this study were expressed as mean ± standard error of mean (SEM). All experiments were performed at least 3 times. Data were analyzed using the unpaired *t*-test to compare the means of 2 groups. *p* < 0.05 was considered statistically significant.

## 3. Results

### 3.1. Characteristics of Bone Marrow and Amniotic Tissue-Derived Mesenchymal Stromal Cells

**Figure 1:**
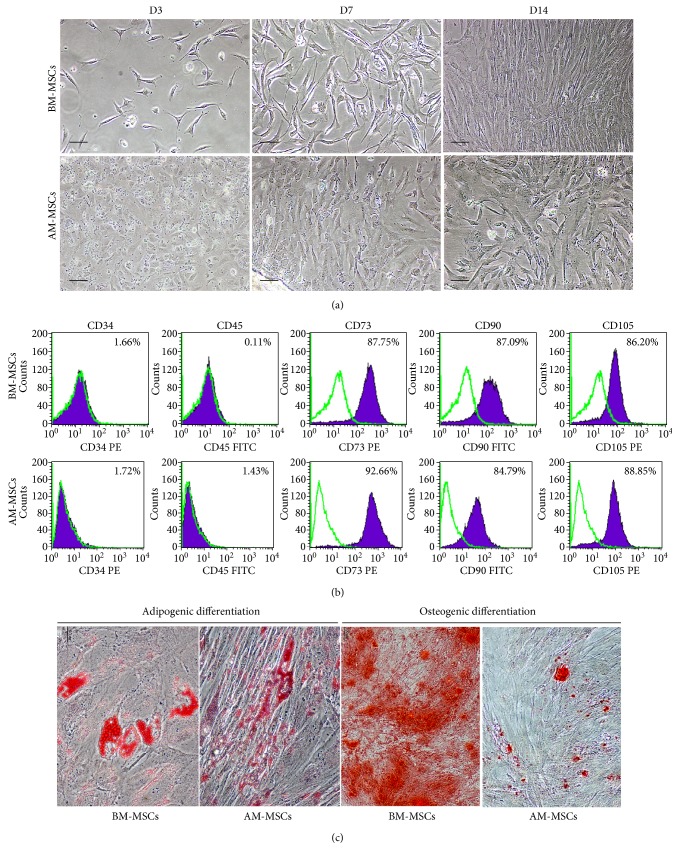
The characteristic of mesenchymal stromal cells derived from amnion (AM-MSCs) and bone marrow (BM-MSCs). (a) The adherent cells exhibited the spindle-shaped morphology and reached 80% confluence at day 14. (b) Immunophenotype of AM-MSCs and BM-MSCs at passage 3. (c) The adipogenic and osteogenic differentiation potential of AM-MSCs and BM-MSCs. The formation of lipid droplet was observed in cytoplasm of AM-MSCs and BM-MSCs after adipogenic induction for 35 and 21 days, respectively. Alizarin red S positive was observed in AM-MSCs and BM-MSCs cultured in osteogenic differentiation medium for 21 and 14 days. Micron bar = 100 *μ*m.

After culture for 3 days, both bone marrow- and amniotic tissue-derived cells attached to the culture surface and displayed fibroblast-like morphology (Figure 1(a)). Those fibroblast-like cells rapidly proliferated, and their density reached 80% confluence within the first two weeks (Figure 1(a)). There was no obvious difference between the morphology of bone marrow- and amniotic tissue-derived MSCs (Figure 1(a)). It is worthy to note that while the bone marrow-derived MSCs (BM-MSCs) could be expanded for only 8–10 passages, the amniotic tissue-derived MSCs (AM-MSCs) could be expanded for at least 20 passages before they reach their replicative senescence.

Both BM-MSCs and AM-MSCs exhibited typical MSC surface markers, being positive for CD73, CD90, and CD105 and being negative for hematopoietic markers, CD34 and CD45. There was no significant difference between the levels of MSC surface marker expressions of AM-MSCs and BM-MSCs (Figure 1(b)).

Both AM-MSCs and BM-MSCs could differentiate to adipocytes and osteoblasts after cultured in adipogenic and osteogenic differentiation media, respectively. After 3 weeks of adipogenic induction, BM-MSCs and AM-MSCs became large cells with lipid droplets that were positive for Oil Red O staining (Figure 1(c)). Moreover, both BM-MSCs and AM-MSCs could also differentiate into osteoblasts after cultured in osteogenic differentiation medium as demonstrated by Alizarin red S staining (Figure 1(c)). Although AM-MSCs possessed an osteogenic differentiation potential, their differentiation potential toward osteoblasts was lesser in degree and took a longer period of time in comparison to BM-MSCs (21 days versus 14 days).

### 3.2. The Effect of BMP-2 on the Expression of Alkaline Phosphatase (ALP) in MSCs

**Figure 2:**
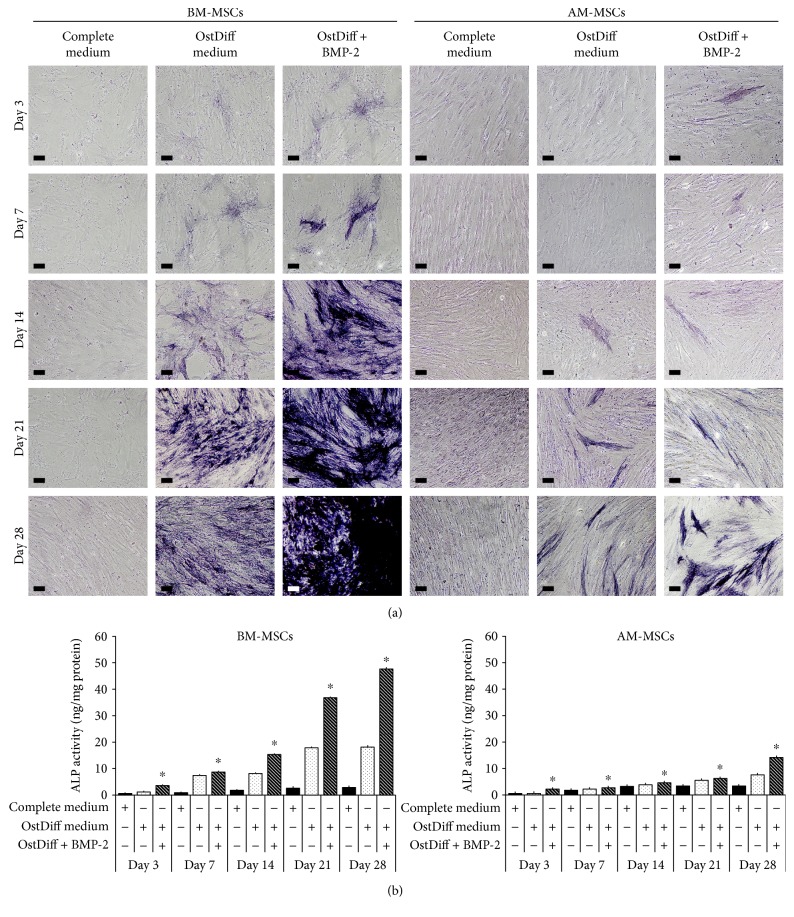
(a) The expression of alkaline phosphatase in AM-MSCs and BM-MSCs cultured in osteogenic differentiation medium supplemented with BMP-2 for 3, 7, 14, 21, and 28 days. (b) Alkaline phosphatase activity of BMP-2 induced osteogenic differentiation of AM-MSCs and BM-MSCs. Data are expressed as mean ± SEM. ^∗^*p* < 0.05 significant difference in comparison to MSCs cultured in osteogenic differentiation medium.

To study the effect of BMP-2 on the osteogenic differentiation of MSCs, both BM-MSCs and AM-MSCs were cultured in osteogenic differentiation medium with or without BMP-2 supplementation for 28 days before their ALP expression was determined by cytochemical staining. The results showed that BMP-2 increased ALP expression in cultured BM-MSCs in comparison to controls (BM-MSCs cultured in osteogenic differentiation medium without BMP-2 supplementation) throughout the entire culture period (Figure 2(a)). The ALP expression of BM-MSCs treated with BMP-2 steadily increased and reached its highest level at the end of culture (culture day 28) (Figure 2(a)). Similar to BM-MSCs, BMP-2 increased ALP expression in cultured AM-MSCs in comparison to controls (AM-MSCs cultured in osteogenic differentiation medium without BMP-2 supplementation) throughout the entire culture period. However, the ALP expression level of AM-MSCs treated with BMP-2 was much lower than that of BM-MSCs cultured under the same condition (Figure 2(a)). Although the ALP expression levels of the BMP-2-untreated groups also steadily increased toward the end of culture, the levels were much lower than those of BMP-2-treated groups (Figure 2(a)).

In agreement with the qualitative cytochemical staining for alkaline phosphatase, the quantitative ALP activity assay confirmed that BMP-2 significantly upregulated the ALP activity in both BM-MSCs and AM-MSCs throughout the entire culture period (Figure 2(b)). The ALP activity of both BM-MSCs and AM-MSCs treated with BMP-2 steadily increased toward the end of culture (culture day 28). However, the ALP activity of AM-MSCs treated with BMP-2 was much lower than that of BM-MSCs cultured under the same condition (Figure 2(b)).

### 3.3. The Effect of BMP-2 on the Expression Levels of Osteogenic Genes

**Figure 3:**
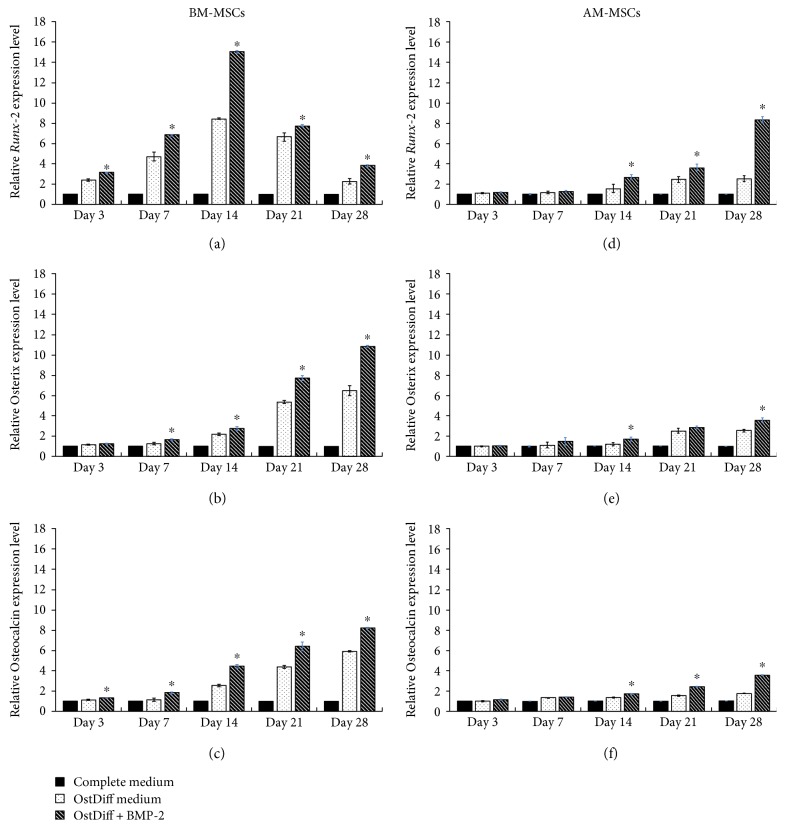
The relative osteogenic gene expression in BMP-2 induced osteogenic differentiation of AM-MSCs in comparison to BM-MSCs. Data are presented as mean ± SEM. ^∗^*p* < 0.05 significant difference compared to MSCs cultured in osteogenic differentiation medium.

To study the effect of BMP-2 on the expression levels of osteogenic gene, the expression levels of several osteogenic genes including runt-related transcription factor 2 (*RUNX-2*), osterix (*OST*), and osteocalcin (*OCN*) in BM-MSCs and AM-MSCs treated with BMP-2 were determined and compared with controls (BMP-2-untreated groups). The results showed that BMP-2 significantly upregulated the expression levels of *RUNX-2*, *OST*, and *OCN* in BM-MSCs at culture days 7, 14, 21, and 28 after osteogenic induction (Figures 3(a), 3(b), and 3(c)). The expression level of *RUNX-2* steadily increased and reached its highest level on culture day 14 before it gradually declined toward the end of culture (Figure 3(a)). Distinct from *RUNX-2*, the expression levels of both *OST* and *OCN* gradually increased throughout the entire culture period and reached their highest points at the end of culture (culture day 28) (Figures 3(b) and 3(c)). Similar to BM-MSCs, BMP-2 significantly upregulated the expression levels of *RUNX-2*, *OST*, and *OCN* in AM-MSCs at culture days 14, 21, and 28 after osteogenic induction (Figures 3(d), 3(e), and 3(f)). The expression levels of *RUNX-2*, *OST*, and *OCN* in AM-MSCs steadily increased throughout the entire culture period and reached their highest points at the end of culture (culture day 28). However, the expression levels of those osteogenic genes in AM-MSCs treated with BMP-2 were much lower than those of BM-MSCs cultured under the same condition. Although the expression levels of osteogenic genes in the BMP-2-untreated groups also increased toward the end of culture, the levels were much lower than those of BMP-2-treated groups (Figure 3).

### 3.4. The Expressions of miR-31, miR-106a, and miR-148a during Osteogenic Differentiation

**Figure 4:**
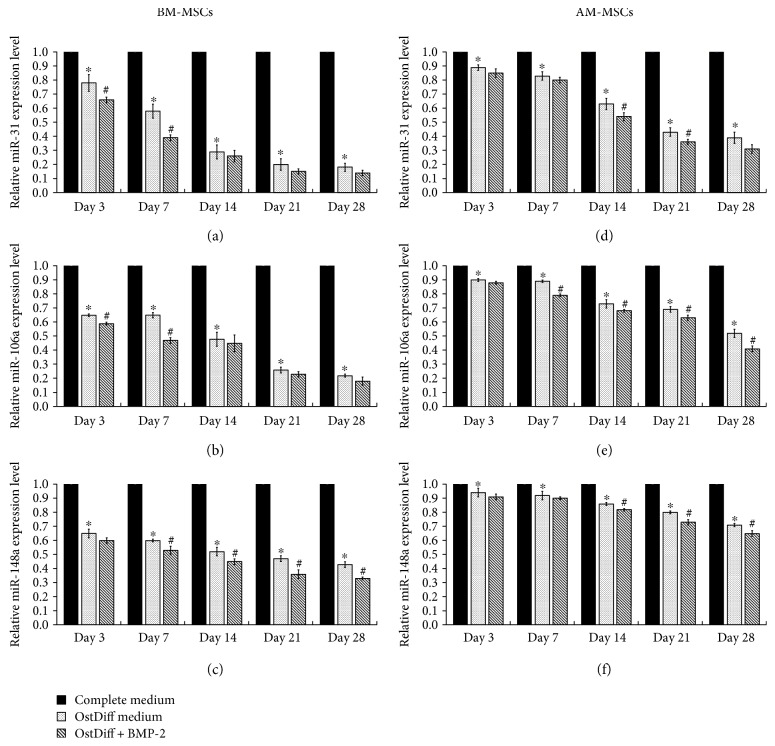
The relative microRNA expression in BM-MSCs (a–c) in comparison to that in AM-MSCs (d–f). Data are presented as mean ± SEM. ^∗^*p* < 0.05 significant difference compared to MSCs cultured in complete medium. ^#^*p* < 0.05 significant difference compared to MSCs cultured in osteogenic differentiation medium.

To explore the alteration of miRNA expression in MSCs during the course of osteogenic differentiation, the expressions of miR-31, miR-106a, and miR-148a were quantified at days 3, 7, 14, 21, and 28 using TaqMan microRNA assays and qRT-PCR. The results showed that the expression of miR-31 was reduced in a time-dependent manner during the process of osteogenic differentiation of BM-MSCs (Figure 4(a)). In addition, the expression of miR-31 in BM-MSCs was significantly decreased after treatment with BMP-2 (Figure 4(a), *p* < 0.05). Similar to BM-MSCs, the expression of miR-31 during the process of osteogenic differentiation of AM-MSCs was also reduced in a time-dependent manner (Figure 4(d)). Interestingly, there was no dramatically change in the expression of miR-31 in AM-MSCs during the first week of osteogenic induction when compared to that of BM-MSCs. Nevertheless, the expression of miR-31 in AM-MSCs was intensely decreased after 14 days of osteogenic induction especially in BMP-2-treated MSCs (Figure 4(d)). Although miR-31 expression was downregulated in AM-MSCs during osteogenic differentiation in the presence or absence of BMP-2, the expression level was still higher than that of BM-MSCs in every time point. Additionally, the expressions of miR-106a and miR-148a were found to be robustly downregulated in BM-MSCs during osteogenic differentiation in the presence or absence of BMP-2 (Figures 4(b) and 4(c)). The expressions of miR-106a and miR-148a during osteogenic differentiation of AM-MSCs were also significantly reduced in a time-dependent manner similar to that of BM-MSCs (Figures 4(e) and 4(f)). However, AM-MSCs showed less reduction in miR-106a and miR-148a expressions when compared to BM-MSCs. It is important to note that AM-MSCs showed the least reduction of miR-106a and miR-148a expressions. These data indicated that miR-31, miR-106a, and miR-148a were downregulated during the process of osteogenic differentiation of both BM-MSCs and AM-MSCs. Fascinatingly, the downregulated level of these miRNAs were in good agreement with osteogenic differentiation potential of these MSCs.

### 3.5. The Expression Levels of MicroRNAs after the Transient Transfection with miRNA Inhibitors

**Figure 5:**
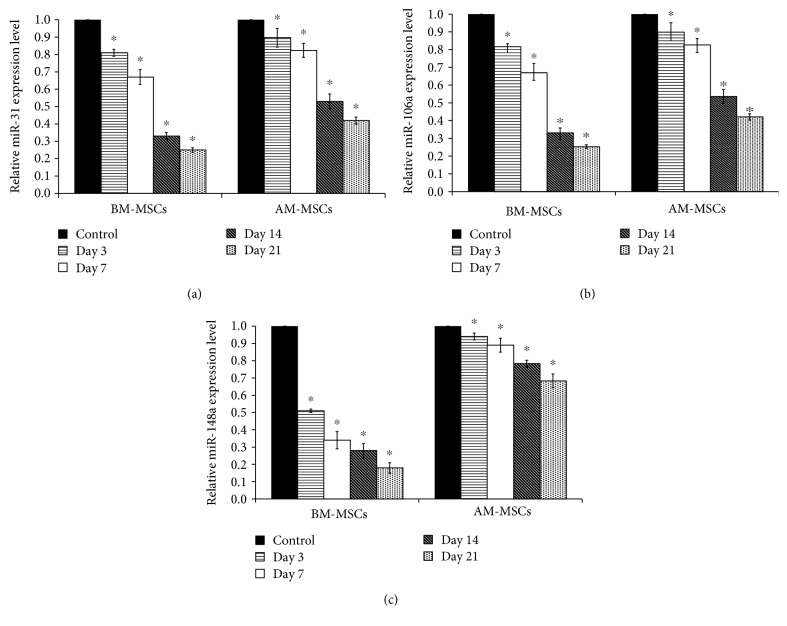
Mean value of relative expressions of miR-31 (a), miR-106a (b), and miR-148a (c) during osteogenic differentiation of AM-MSCs and BM-MSCs after the transient transfection with anti-miR-31, anti-miR-106a, and anti-miR-148a, respectively. Data are presented as mean ± SEM. ^∗^*p* < 0.05 significant difference compared to MSCs cultured in osteogenic differentiation medium + 10 nM negative anti-miR.

To determine the effect of miRNA on osteogenic differentiation capacity of MSCs, BM-MSCs and AM-MSCs were transfected with anti-miR-31, anti-miR-106a, and anti-miR-148a. After induced differentiation, the expression levels of miR-31, miR-106a, and miR-148a were quantified by qRT-PCR at cultured days 3, 7, 14, and 21. The result demonstrated that after transfected with anti-miR-31, the expression of miR-31 was reduced in a time-dependent manner during the process of osteogenic differentiation of BM-MSCs when compared to that transfected with negative miRNA. Similar to BM-MSCs, the expression of miR-31 during the process of osteogenic differentiation of AM-MSCs was also reduced in a time-dependent manner (Figure 5(a)). The expression of miR-31 in these MSCs was intensely decreased after 14 days of osteogenic induction especially in AM-MSCs. Although miR-31 expression was downregulated in AM-MSCs during osteogenic differentiation in the presence of anti-miR-31, the expression level was still higher than that of BM-MSCs in every time point. Additionally, the expressions of miR-106a and miR-148a were found to be robustly downregulated in BM-MSCs during osteogenic differentiation in the presence of anti-miR-106a and anti-miR-148a (Figures 5(b) and 5(c)). The expressions of miR-106a and miR-148a during osteogenic differentiation of AM-MSCs were also significantly reduced in a time-dependent manner similar to those of BM-MSCs. In addition, there was a dramatical change in the expressions of miR-106a in AM-MSCs and miR-148a in BM-MSCs along the process of osteogenic induction when compared to that of negative control (*p* < 0.05). However, AM-MSCs showed less reduction in miR-106a and miR-148a expressions when compared to BM-MSCs. Although miRNA expression levels decreased over time in 3 anti-miRNAs transfection groups, the miRNA expression level in AM-MSCs was significantly higher than that in BM-MSCs at every time point examined (Figure 5).

### 3.6. The Expression of Alkaline Phosphatase in MSCs after Treated with miRNA Inhibitors

**Figure 6:**
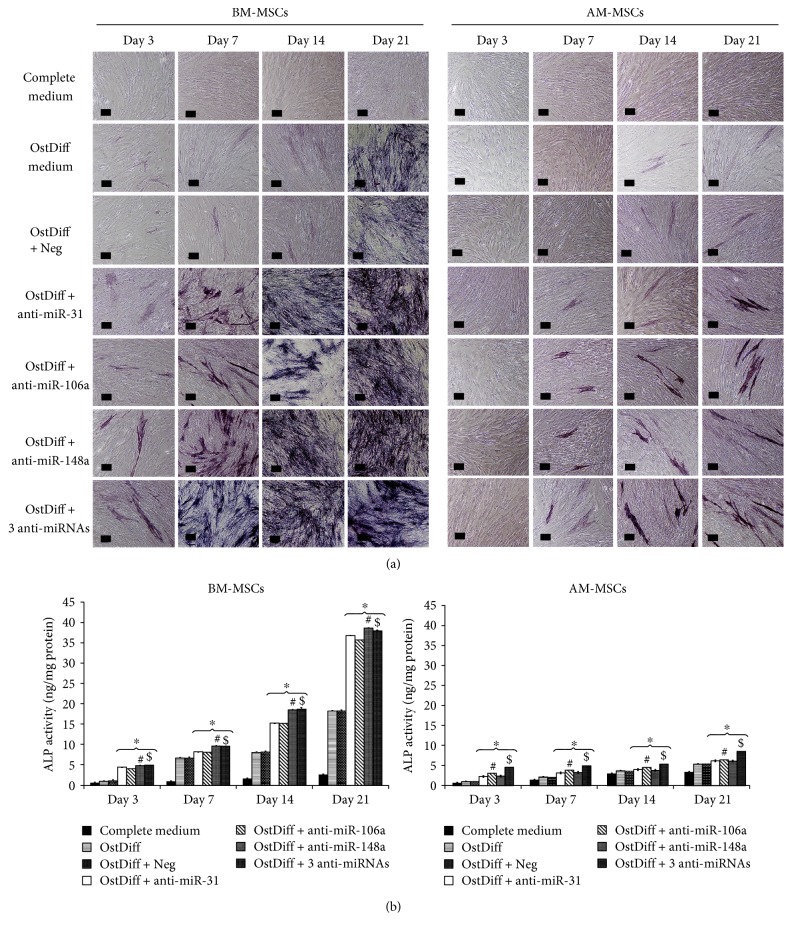
(a) ALP staining in AM-MSCs and BM-MSCs after treated with anti-miR-31, anti-miR-106a, and anti-miR-148a for 3, 7, 14, and 21 days. (b) Alkaline phosphatase activity of AM-MSCs and BM-MSCs after transient transfection with miRNA inhibitors. Data are presented as mean ± SEM. ^∗^*p* < 0.05 significant difference compared to MSCs cultured in osteogenic differentiation medium + negative control. ^#,^^$^*p* < 0.05 significant difference compared to BM-MSCs cultured in osteogenic differentiation medium + anti-miR-31 or anti-miR-106a or AM-MSCs cultured in osteogenic differentiation medium + anti-miR-31 or anti-miR-148a.

To investigate the roles of miR-31, miR-106a, and miR-148a in osteogenic differentiation of AM-MSCs in comparison to that of BM-MSCs, the inhibitors of miR-31, miR-106a, and miR-148a were employed to alter the expressions of miR-31, miR-106a, and miR-148a. The expressions of ALP were examined in MSCs after transfection for 3, 7, 14, and 21 days. The results revealed that the inhibitions of each miRNAs, miR-31, miR-106a, and miR-148a, and the inhibition using a combination of 3 anti-miRNAs enhanced the expression of ALP in both BM-MSCs and AM-MSCs compared to MSCs cultured in osteogenic differentiation medium without miRNA inhibitor and MSCs cultured in osteogenic differentiation medium with negative control miRNA (Figure 6(a)). Nevertheless, combination of the 3 anti-miRNAs did not reveal the dramatical change in ALP expression in both BM-MSCs and AM-MSCs (Figure 6(a)). In addition, ALP expression in AM-MSCs after treated with miRNA inhibitors was lower than that in BM-MSCs in every time points.

The activities of intracellular ALP in BM-MSCs and AM-MSCs transfected with anti-miR31, anti-miR-106a, anti-miR-148a, and the combination of these anti-miRNAs were also quantitatively assessed using colorimetric enzymatic assay at days 3, 7, 14, and 21. The results demonstrated that the BM-MSCs cultured in osteogenic differentiation medium with 10 nM anti-miR-148a showed a clear dominance compared to the others (Figure 6(b)). As early as 7 days after transfection with anti-miR-31, anti-miR-106a, and anti-miR-148a, or the combination of 3 anti-miRNAs, BM-MSCs had about a fold increase in ALP activity. Interestingly, the activity of ALP in the anti-miRNA-treated groups significantly increased with time. At days 14 and 21, ALP activities in anti-miRNA-transfected groups were significantly increased up to 2-fold, compared with MSCs cultured in osteogenic differentiation medium without anti-miRNA and osteogenic differentiation medium added with a negative control of anti-miRNA (*p* < 0.05). Remarkably, the transfection with anti-miR-31, anti-miR-106a, and anti-miR-148a, or the combination of 3 anti-miRNAs, induced less than a fold increase in ALP activity in AM-MSCs compared with untreated osteogenic differentiation culture (*p* < 0.05). Similar to ALP staining, although ALP activity increased over time in anti-miRNA-transfected groups, the ALP activity in AM-MSCs was significantly less than that in BM-MSCs at every time point examined (Figure 6(b)).

### 3.7. The Expression of Osteogenic Lineage Genes in MSCs after the Transient Transfection with miRNA Inhibitors

**Figure 7:**
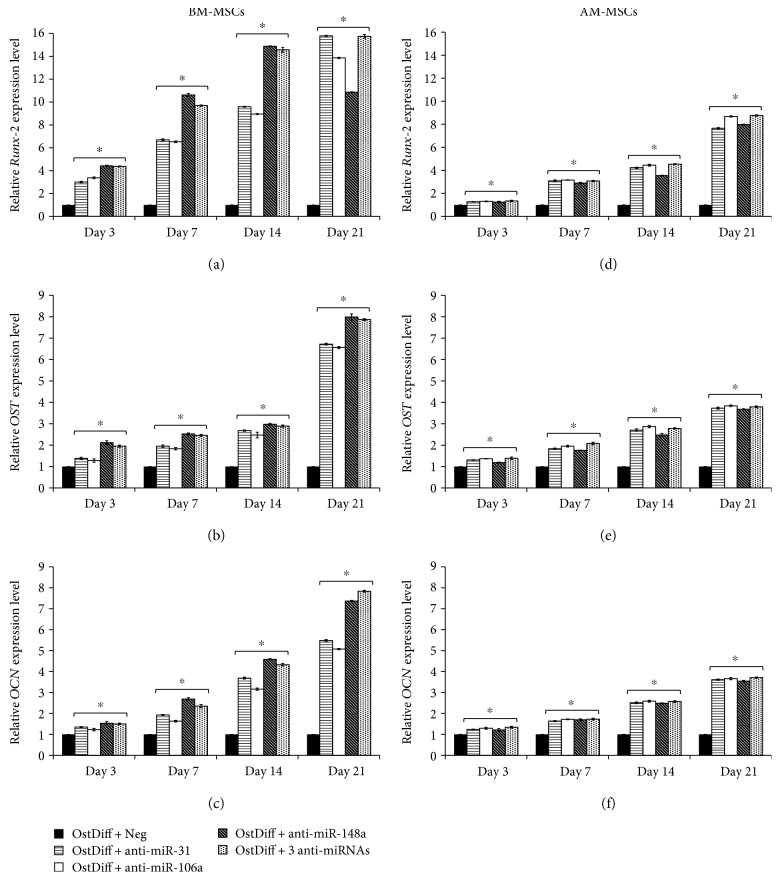
The relative osteogenic gene expression in BM-MSCs (a–c) and AM-MSCs (d–f), after the transient transfection with anti-miR-31, anti-miR-106a, and anti-miR-148a and the combination of 3 anti-miRNAs. Data are presented as mean ± SEM. ^∗^*p* < 0.05 significantly different compared to MSCs cultured in osteogenic differentiation medium + negative anti-miR.

The effects of miRNA inhibitor on osteogenic differentiation potential of BM-MSCs and AM-MSCs were further investigated through gene expression analysis including *RUNX-2*, *OST*, and *OCN* following 3, 7, 14, and 21 days of culture. The results demonstrated that after transfected with anti-miR-31, the expressions of *RUNX-2*, *OST*, and *OCN* were increased in a time-dependent manner during the process of osteogenic differentiation of BM-MSCs when compared to those transfected with negative anti-miRNA (Figures 7(a), 7(b), and 7(c)). The expression of *RUNX-2* in BM-MSCs was increased over time from day 3 to day 14. The peak of *RUNX-2* expression was found during day 14 in BM-MSCs cultured in osteogenic differentiation medium added with anti-miR-148a (Figure 7(a)). Nevertheless, BM-MSCs cultured in osteogenic differentiation medium with these anti-miRNAs showed significantly higher *RUNX-2* expression than those cultured in osteogenic differentiation added with a negative control of anti-miRNA (Figure 7(a)). Similar to BM-MSCs, *RUNX-2* expression was increased over time from day 3 to day 21 in AM-MSCs cultured in osteogenic differentiation medium added with anti-miR-31, anti-miR-106a, anti-miR-148a, and the combination of 3 anti-miRNAs. Interestingly, AM-MSCs treated with these anti-miRNAs showed higher expression of *RUNX-2* than those of untreated groups (Figure 7(d)). The effects of miRNA inhibitors on the expression levels of other osteogenic lineage genes in AM-MSCs were also different from BM-MSCs. The expression of *OST* was increased over time from day 3 to day 21 in both BM-MSCs and AM-MSCs cultured in osteogenic differentiation medium added with anti-miR-31, anti-miR-106a, anti-miR-148a, and the combination of 3 anti-miRNAs (Figures 7(b) and 7(e)). The transfections with anti-miR-31, anti-miR-106a, anti-miR-148a, and the combination of 3 anti-miRNAs significantly upregulated the expression of *OST* in AM-MSCs on days 3, 7, 14, and 21 of culture (Figure 7(e)) similar to that in BM-MSCs. Nevertheless, BM-MSCs treated with miRNA inhibitors had significantly higher *OST* expression than AM-MSCs especially at day 21. Similar to *OST*, minimum *OCN* expression was detected in BM-MSCs at day 3. The *OCN* expression levels were significantly increased in BM-MSCs transfected with anti-miR-31, anti-miR-106a, anti-miR148a, and the combination of 3 anti-miRNAs at days 3, 7, 14, and 21, compared with the negative controls of anti-miRNA (Figure 7(c)). The expressions of *OCN* were increased to the same extent with time in AM-MSCs in anti-miR-31, anti-miR-106a, anti-miR-148a, and the combination of 3 anti-miRNAs treated groups (Figure 7(f)). However, the upregulations of *OCN* in AM-MSCs were dramatically observed in anti-miR-31, anti-miR-106a, anti-miR-148a, and the combination of 3 anti-miRNAs transfected groups at days 14 and 21 (Figure 7(f)).

## 4. Discussion

Human MSCs are multipotent cells which presented in the bone marrow, adipose tissue, and postnatal tissues including amnion [17–19]. MSCs have been regarded as a potential source for cell therapy due to their property to promote tissue repair [20, 21]. The approach for enhancing osteogenic differentiation is one of the most crucial issues in bone tissue regeneration [20, 22]. Bone morphogenetic protein 2 (BMP-2) is probably the most important growth factor in bone formation both *in vitro* and *in vivo* [4, 23, 24]. A previous study reported that BMP-2 could stimulate the osteogenic differentiation of MSCs [5, 25]. Currently, most of the MSCs used in clinical field and preclinical study are BM-MSCs. Nonetheless, bone marrow aspiration is an invasive procedure. Of the most important, BM-MSCs have a limited number in the bone marrow [26, 27]. Recent study reported that MSCs could be isolated from amniotic tissue without hurt to both a mother and an infant [2, 3]. AM-MSCs showed the similar characteristics to BM-MSCs; however, the differences including osteogenic differentiation potential were also reported [4, 28]. AM-MSCs use a longer period of time for differentiation into osteoblasts compared to BM-MSCs [3]. Therefore, the strategy for enhancing osteogenic differentiation capability of AM-MSCs is needed. This study revealed that MSCs isolated from amnion had the characteristic of MSCs according to the criteria of the International Society for Cellular Therapy [1]. The plastic-adherent cells from amnion exhibited fibroblast-like morphology similar to that of BM-MSCs. They were positive for typical MSC markers including CD73, CD90, and CD105 and negative for hematopoietic markers including CD34 and CD45. These evidences are in harmony with the previous studies [1, 29, 30]. In addition, AM-MSCs could differentiate into adipogenic and osteogenic lineages similar to BM-MSCs. However, AM-MSCs took a longer period of time than BM-MSCs for differentiation into both adipogenic and osteogenic lineages.

This study investigated the effect of BMP-2 on osteogenic differentiation of AM-MSCs compared to that of BM-MSCs using ALP staining and ALP activity assay. The results demonstrated that both BM-MSCs and AM-MSCs had higher degree of ALP expression after BMP-2 treatment. Harmoniously, the expressions of osteogenic genes were also increased in both BM-MSCs and AM-MSCs after BMP-2 treatment. These evidences are in coherence with the previous study which demonstrated that BMP-2 increased the expressions of ALP and osteogenic genes in MSCs derived from the umbilical cord [4]. However, the mechanisms involved in BMP-2-mediated osteogenesis are not fully implicit. Several studies demonstrated that BMP-2 is an important growth factor for osteogenic differentiation [5, 20, 24]. BMP-2 regulates the expression of target genes which are involved in osteoblast differentiation [31]. In this study, the osteogenic genes, including *RUNX-2, OST*, and *OCN*, were upregulated in BM-MSCs after BMP-2 treatment. Interestingly, the highest level of *RUNX-2* expression was observed in BM-MSCs at day 14. In contrast, the highest expression of *RUNX-2* in AM-MSCs was observed at day 28. RUNX-2 is one of the earliest master transcription factors that direct the differentiation of MSCs into osteoblasts [8]. Prior study suggested that RUNX-2 plays an important role in the commitment step for osteogenic differentiation [32]. While BMP-2 could increase the osteogenic differentiation potential of AM-MSCs, the outcome is less noticeable compared to BM-MSCs. This might be due to the endogenous difference between BM-MSCs and AM-MSCs.

Several studies have demonstrated that miRNAs were associated with stem cell self-renewal and differentiation. They play a key role in controlling stem cell activities [15, 33]. To determine the role of miRNA on osteogenic differentiation of MSCs, the expression of miRNA during osteogenic differentiation of AM-MSCs and BM-MSCs was quantified using qRT-PCR. The results demonstrated that the expressions of miR-31, miR-106a, and miR-148a were downregulated during osteogenic differentiation of both BM-MSCs and AM-MSCs. In addition, the expressions of these miRNAs were also decreased after BMP-2 treatment in both BM-MSCs and AM-MSCs. After transfection with anti-miRNAs, ALP activity and the expression of osteogenic markers were increased in AM-MSCs similar in BM-MSCs. Although the downregulation of these miRNAs could enhance the osteogenic differentiation capacity of AM-MSCs, the effect was less pronounced than that of BM-MSCs. This might be due to the endogenous difference in osteogenic differentiation capacity between BM-MSCs and AM-MSCs. Nevertheless, this study demonstrates that transfection of anti-miRNAs could enhance the osteogenic differentiation capacity of both BM-MSCs and AM-MSCs as evidenced by increased ALP expression and osteogenic gene expressions. Recent study revealed that miR-31, miR-106a, and miR-148a were underexpressed during osteogenic differentiation of human BM-MSCs. The putative targets of these miRNAs, predicted by bioinformatics analysis, include *RUNX-2*, *CBFB*, and BMPs which are involved in bone formation [15]. A previous study reported that miR-31 is a negative regulator of osteogenic differentiation. MiR-31 repressed osteogenesis of human MSCs by decreased expression levels of *RUNX-2*, *OCN*, and *OST* [34]. In accordance with this study, the anti-miR-31-modified adipose tissue-derived stem cells via lentiviral vector were applied to repair critical-sized defects (CSDs) in rats combined with the *β*-tricalcium phosphate (*β*-TCP) scaffolds. Micro-CT displayed that miR-31 knockout can improve ossification *in vivo* [35]. Moreover, BMP-2 is inhibited by miR-106a which may suppress osteogenesis through the BMP/*RUNX-2* pathway [36].

In conclusion, this study reported the effects of BMP-2 and miRNAs on osteogenic differentiation of AM-MSCs and BM-MSCs. The results demonstrated that AM-MSCs could be isolated and easily expanded in culture. BMP-2 enhanced osteogenic differentiation of AM-MSCs similar to BM-MSCs by upregulating the expression of osteogenic genes including *RUNX-2*, *OST*, and *OCN*. The ALP activity as well as osteogenic gene expressions was increased during osteogenic differentiation of BM-MSCs and AM-MSCs. In contrast to expression of osteogenic genes, the expressions of miR-31, miR-106a, and miR-148a were decreased during osteogenic differentiation. The transient transfection with anti-miR-31, anti-miR-106a, and anti-miR-148a could increase ALP activity and the expression of osteogenic genes of both BM-MSCs and AM-MSCs. The knowledge gained from this study increases understanding on the mechanisms underlying the effects of BMP-2 and miRNAs on osteogenic differentiation of both AM-MSCs and BM-MSCs. In addition, this study further revealed the possibility of combining miRNA and BMP-2 for synergistic *in vitro* osteogenic induction which might led to the progress of bone regeneration using MSCs.

## Figures and Tables

**Table 1:** The primers and the product size.

Gene	Forward primer	Reverse primer	Product size (bp)
*RUNX-2*	5′-GACAGCCCCAACTTCCTGT-3′	5′-CCGGAGCTCAGCAGAATAAT-3′	159
*Osterix*	5′-TGCTTGAGGAGGAAGTTCAC-3′	5′-CTGCTTTGCCCAGAGTTGTT-3′	114
*Osteocalcin*	5′-CTCACACTCCTCGCCCTATT-3′	5′-TCAGCCAACTCGTCACAGTC-3′	245
*GAPDH*	5′-CAATGACCCCTTCATTGACC-3′	5′-TTGATTTTGGAGGGATCTCG-3′	159

**Table 2:** The mature miRNA sequence.

miRNA	Mature miRNA sequence
hsa-miR-31-5p	AGGCAAGAUGCUGGCAUAGCU
hsa-miR-106a-5p	AAAAGUGCUUACAGUGCAGGUAG
hsa-miR-148a-5p	AAAGUUCUGAGACACUCCGACU
U6	GTGCTCGCTTCGGCAGCACATATACTAAAATTGGAACGATACAGAGAAGATTAGCATGGCCCCTGCGCAAGGATGACACGCAAATTCGTGAAGCGTTCCATATTTT
